# Concentrations of heavy metals and biogenic elements in needles of Norway spruce and Scots pine from Polish peatlands

**DOI:** 10.1038/s41598-026-51610-y

**Published:** 2026-05-19

**Authors:** Norbert Szymański, Stanisław Łyszczarz, Michał Jasik, Wojciech Piaszczyk, Andrzej Szlachta, Mirosław Żelazny, Ewa Błońska, Jarosław Lasota, Stanisław Małek

**Affiliations:** 1 Department of Ecology and Silviculture, Faculty of Forestry, University of Agriculture in Krakow, 29 Listopada 46, 31-425 Kraków, Poland; 2 Department of Hydrology, Institute of Geography and Spatial Management, Jagiellonian University, Gronostajowa 3a, 30-387 Kraków, Poland

**Keywords:** Heavy metals, Biogenic elements, Histosol type, Bioaccumulation, Needles, Peatlands, Biogeochemistry, Ecology, Ecology, Environmental sciences

## Abstract

The study characterised the content and spatial variability of heavy metals and selected biogenic elements (Cd, Co, Cr, Cu, Ni, Pb, Zn, Al, Ca, Fe, K, Mg, Mn, Na, P, N, C) in one-year-old needles of Norway spruce and Scots pine from 262 peatlands in Poland in the light of previous studies and norms, and to determine the relationship between the content of elements in needles and the types of Histosol (fibric, hemic, sapric). The spatial variation in heavy metal content in needles is not related to southern regions of Poland with high human footprint index which suggests that episodic local sources of pollution and transboundary transport of air pollutants are likely to influence their bioaccumulation. It is also not related to the type of Histosol or the pH of water and soil, which indicates that heavy metals accumulate mainly passively from the atmosphere. Despite exceeding the optimal values for some elements, heavy metal levels do not pose a toxicological risk. Elements such as chromium in spruce and cadmium in pine can serve as markers of the impact of industrial emissions on peatland ecosystems. The concentrations of the most studied elements in Poland were lower than in other European countries.

## Introduction

Peatlands are among the most valuable and, at the same time, the most vulnerable terrestrial ecosystems. They play a significant role in regulating hydrological conditions, storing carbon, and shaping local and regional biodiversity^1,2^. Due to their specific characteristics – high moisture, acidic conditions, and a high content of organic matter – they also constitute environments capable of accumulating various chemical elements, including both essential biogenic elements and potentially toxic heavy metals^3^.

In recent decades, an increase in human footprint index on the natural environment has been observed, mainly associated with industrial development, transportation, and agricultural intensification. As a consequence of these processes, emissions of pollutants into the atmosphere have increased, followed by their deposition in terrestrial ecosystems, including peatlands^4^. Due to the limited dynamics of matter circulation and specific geochemical conditions, peatlands may function as long-term reservoirs of heavy metals, making them particularly important objects of study in environmental chemistry^5^.

Poland has been one of the most polluted European countries with the worst state of forest ecosystems in the last half-century. The pollution of flora is the result of air pollution with heavy metals, which is mainly related to the history of heavy industry development, especially metallurgy and mining in southern Poland, northern Czech Republic and Slovakia, but also to the development of the energy, construction, transport, municipal and housing sectors. During the socialist economy (1945-1989), heavy metal and dust emissions were very high due to the lack of effective filtration systems and lax environmental standards. With the development of the capitalist economy after 1989, many harmful factories were closed or modernised. After Poland’s accession to the European Union in 2004, stricter emission standards were introduced, leading to a significant decrease in heavy metal and dust emissions into the air compared to 20th-century levels^6–8^.

Heavy metals travel long distances with air and dust. They settle on plant surfaces and soil through rainfall and dust deposition^9^. As the pH of the soil and water decreases, they become more available to plants because they dissolve more easily and are absorbed more quickly by the roots. Removing heavy metals from the soil is time-consuming, so plants absorb them in excess for many years, even if air pollution has decreased^10^.

Norway spruce (*Picea abies* (L.) H. Karst.) has been successfully used as a bioindicator for monitoring heavy metal pollution in European forests^3,11–17^, as well as Scots pine (*Pinus sylvestris* L.)^3,18–25^. However, there are no current results of biomonitoring studies of forest vegetation covering the entire territory of Poland, which gave rise to their implementation on the example of spruce and pine growing on peatlands. These areas and connected with them organic soils (Histosols) spread on 4% Poland^26^. Histosols accumulate and filter pollutants, and focus organic carbon, and constitute habitat of many specific organisms^27^. Diagnosing the health status of vegetation and actively protecting these wetland areas is essential for maintaining and even increasing the retention capacity of the environment in the context of increasingly rapid changes in meteorological conditions throughout the year and the occurrence of droughts and floods^28^.

Based on the analysed literature, the following hypotheses were put forward: (1) higher heavy metal contents are found in the needles of spruce and pine trees growing in the southern half of Poland, where is higher human footprint index than northern Poland; (2) the heavy metal content in the needles of the studied species does not exceed the permissible norms and is lower than that reported in earlier literature; (3) higher element contents are found in the needles of spruce and pine trees growing on Fibric Histosol with low soil and water pH than on Sapric Histosol with high pH values. In view of the above, the aim of the study was to characterise the content and spatial variability of heavy metals and selected biogenic elements in the needles of Norway spruce and Scots pine from peatlands in Poland in the light of previous studies and norms, and to determine the relationship between the content of elements in the needles and the types of Histosol.

The obtained results may contribute significantly to the understanding of peatland functioning under human impact and provide data useful for environmental monitoring and the planning of conservation measures. They gain the knowledge about element cycling in ecosystems and the impact of human activity on the natural environment.

## Materials and methods

**Fig. 1:**
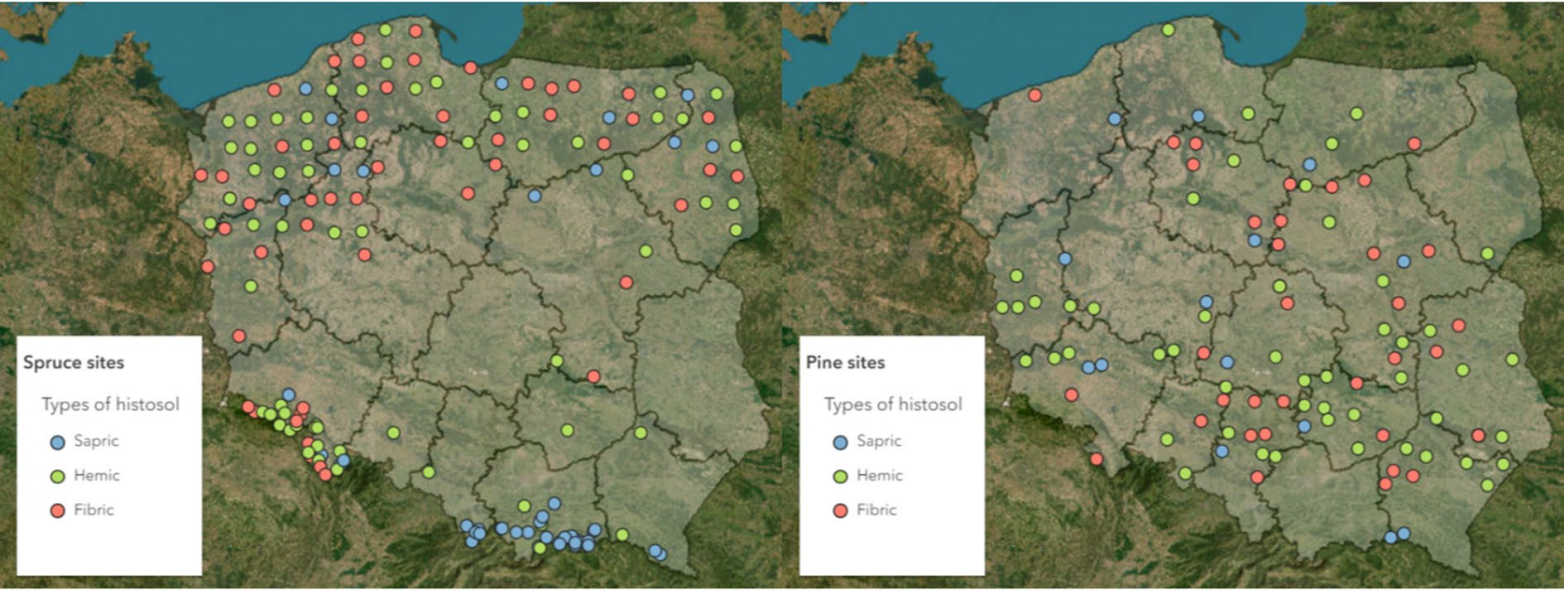
Distribution of spruce and pine needle sampling points in relation to Histosol type (the basemap is from ArcGIS Online – version February 2026, https://www.arcgis.com/).

In the uplands and lowlands of Poland, a grid of points was laid out at regular intervals of 30 km × 30 km, while in the mountains, the grid was laid out at intervals of 10 km × 10 km. The needles were collected from the peatlands closest to the centre of the grid. The locations of the peatlands were determined on the basis of the Map of peatlands of the General Directorate for Environmental Protection in 2024. Annual precipitation was from 500 mm (lowlands) to 1700 mm (mountains) and climatic water balance was from -380 mm (lowlands) to +780 mm (mountains) (https://klimat.imgw.pl/). From each peatland, five subsamples was taken from the tree closest to the sampling point of soil (Histosols) and water. Histosols cover on sandy and sandy-clay glacial formations (northern Poland), flow sand formations in river valleys, lake sediments – gyttjas and lacustrine chalks in outflowless depressions (center Poland), rock or wasteland in marshes and spring (mountain)^29^.Three types of organic materials were in Histosols (fibric, hemic, sapric). Fibric material consists more than 2/3 (by volume) dead plant tissues, hemic contains from 1/6 to 4/6, sapric contains below 1/6^27,30^. The composite sample was stored in paper bag. A total of 162 samples of one-year-old spruce needles and 100 samples of one-year-old pine needles were collected in September 2024 (Fig. 1). The needles were air-dried, homogenised by ginder, and analysed in the Laboratory of Geochemistry of the Forest Environment and Areas Designated for Reclamation (https://labgeochemia.urk.edu.pl/). Heavy metals toxic to trees (Cd, Co, Cr, Pb) and selected elements with a beneficial effect on the life processes of trees, the so-called biogenic elements (Al, Ca, Fe, K, Mg, Mn, Na, P, N, C, Cu, Ni, Zn) were analyzed^31^. C and N contents were measured with an elemental analyser (LECO CNS TrueMac Analyser, Leco, St. Joseph, MI, USA). The Cd, Co, Cr, Cu, Ni, Pb, Zn, Al, Ca, Fe, K, Mg, Mn, Na, P contents were measured with an inductively coupled plasma (ICP) spectrometer (ICP-OES Thermo iCAP 6500 DUO, Thermo Fisher Scientific, Cambridge, U.K). The P concentrations were assessed after the samples underwent digestion in a 1:3 mixture of NHO_3_ and HClO_4_ (reverse aqua regia). This process was carried out using the ETHOS UP microwave system (Milestone), which features an integrated monitoring module that provides real-time feedback on process conditions and automatically stores the complete operational log to support quality control. To ensure that the ICP measurements was both effective and consistent, each analytical batch included an internal standard from proficiency testing (PT) and interlaboratory comparisons (ILC) with Wageningen University & Research (WPAL). All procedures were conducted using spectrally pure nitric and perchloric acids. The pH values of soil and water samples collected near the tree were measured using a potentiometric method in the laboratory. The detailed methodology was described by Piaszczyk et al.^32,33^.

The chemical element contents in the needles were tabular compared to norms defined by Kabata-Pendias^3^ and European background (previous results)^11–25^. The normality of the data distribution was verified using the Shapiro–Wilk test, while the homogeneity of variance was verified using the Levene test. Due to the non-normality of the data distribution, differences between the contents of heavy metals and biogenic elements in needles were analysed using non-parametric Mann–Whitney and Kruskal–Wallis tests. Spearman’s non-parametric rank correlation analysis and principal component analysis (PCA) were used to assess the relationship between heavy metal and biogenic element contents in needles and soil and water pH. This statistical analysis were used to assess influence of human activity on bioaccumulation – the relationship between the chemical element content and the density index of roads, railways, power users, human population, agriculture lands, infrastructure, and also human footprint index in 2024 (weighted sum of the previous indices) from the map with resolution of 300 m^34^, https://wcshumanfootprint.org/). Statistical analyses were performed using STATISTICA 13 software (https://www.statsoft.pl/en/). ArcGIS Online software was used for spatial analyses (https://www.arcgis.com/).

## Results

The mean and maximum contents of the elements studied in this work in spruce needles from the peatlands of Poland were lower than the mean and maximum calculated on the basis of other sites in Europe (Table 1, Fig. 1). The exception is Cu, whose content in needles at eleven sites located in the Wielkopolska and Masurian Lake District, the south part of Mazovian Lowland, and the Żywiec Beskids (here max) was higher than the European maximum. The mean and maximum heavy metal content in needles in peatlands in Poland did not exceed the toxicity norms, except for Cr at one site in the Żywiec Beskids (9.73 mg∙kg^−1^). The Ni content found in needles at the site in the Żywiec Beskids (7.61 mg∙kg^−1^) and the Wielkopolska Lake District (5.85 mg∙kg^−1^) was above the normal (optimal) level given in the literature.

**Table 1:** Content of selected chemical elements (mg∙kg^−1^) in one-year-old needles of Norway spruce from peatlands in Poland compared to sites in Europe^a^ and norms for plants^b^.

Chemical element	Poland				Europe				Norms	
	Mean	Min	Max	Sd	Mean	Min	Max	Sites	Normal level	Toxic level
Cd	0.16	0.01	3.69	0.31	0.35	0.07	1.02	24	0.01–5	5–30
Co	0.16	0.01	1.78	0.18	–	–	–	–	0.02–1	15–50
Cr	0.59	0.16	**9.73**	0.76	–	–	–	–	0.1–0.5	**5**–**30**
Cu	5.36	1.31	**18.62**	3.14	6.16	1.88	**11.90**	34	2–30	20–100
Pb	0.26	0.01	0.67	0.11	5.18	0.60	28.80	29	3–10	30–300
Ni	1.78	0.37	**7.61**	1.06	–	–	–	–	**0.1–5**	10–100
Zn	24.96	9.11	47.35	6.67	53.71	16.40	254.00	36	10–150	100–400
Al	32.29	7.24	101.03	15.69	99.12	68.90	140.00	4	–	–
Ca	2499.22	659.36	7438.84	843.14	6391.06	3220.00	10,546.00	15	–	–
Fe	34.46	19.14	84.17	8.83	93.93	10.65	215.00	14	–	–
K	5916.06	2484.09	10,842.23	1763.01	6735.56	4900.00	9343.00	15	–	–
Mg	680.71	424.00	1261.22	127.92	1064.95	273.00	1820.00	16	–	–
Mn	286.92	28.62	912.05	186.32	692.14	57.00	1295.00	14	–	–
Na	20.56	6.64	83.60	11.62	94.41	27.00	261.00	9	–	–
P	1419.77	148.00	2820.03	455.53	1491.63	1130.00	2120.00	15	–	–
C [%]	46.84	44.21	49.30	0.74	–	–	–	–	–	–
N [%]	1.17	0.67	2.12	0.25	–	–	–	–	–	–

^a^^11–16^, ^b^^3^.

Significant values are in bold.

**Fig. 2:**
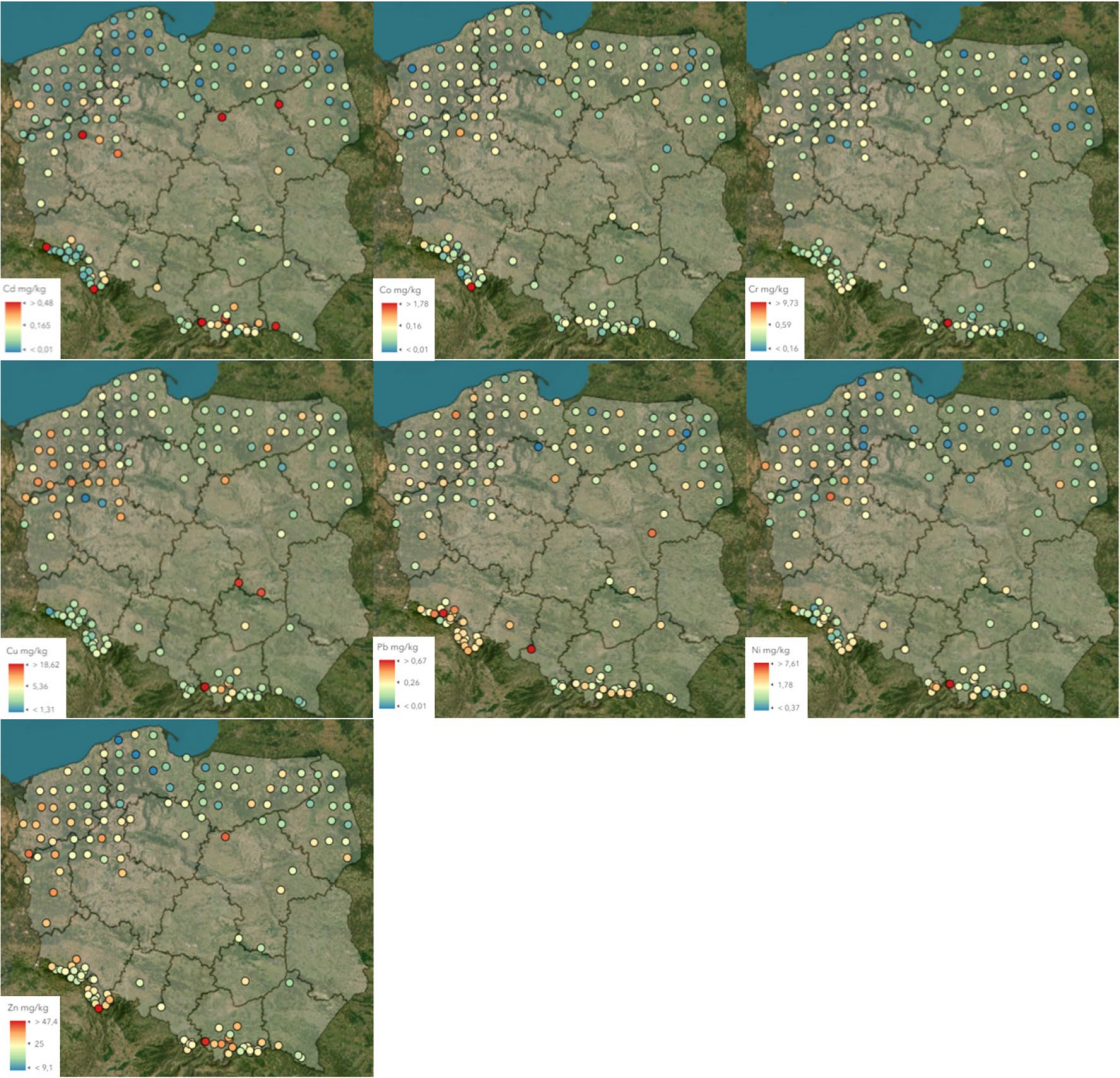
Heavy metal content (mg∙kg^−1^) in one-year-old needles of Norway spruce in Polish peatlands. The beginning of the range –minimum, the middle – mean, the end – maximum value (the basemap is from ArcGIS Online – version February 2026, https://www.arcgis.com/).

**Fig. 3:**
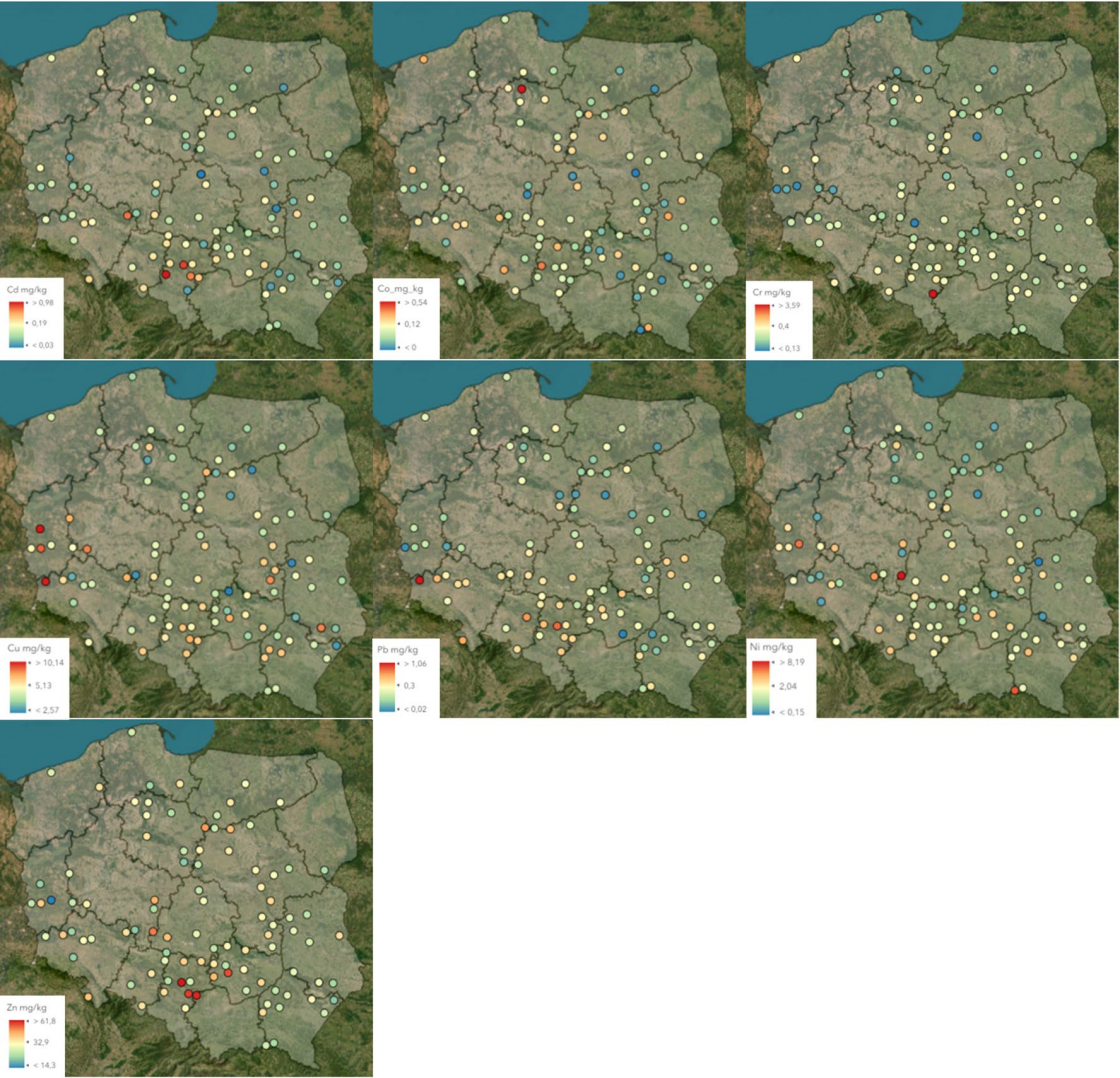
Heavy metal content (mg∙kg^−1^) in one-year-old needles of Scots pine from peatlands. The beginning of the range – minimum , the middle – mean, the end – maximum value (the basemap is from ArcGIS Online – version February 2026, https://www.arcgis.com/).

The spatial variability of heavy metal content in the needles of both spruce and pine did not have a distinct regional character (Figs. 2, 3). The highest values were found in isolated, dispersed locations in Poland. Only in the case of Cu in spruce needles can it be said that the north-eastern part of Poland is characterised by high values of this element (Fig. 2). The heavy metal and biogenic element content in spruce and pine needles is not clearly related to the components of human footprint index (Table 3).

The highest Cd concentrations in spruce needles were found in the Wielkopolska Lake District, the north part of Mazovian Lowlands, the Żywiec Beskids, Niski Beskids and the Eastern Sudetes (maximum 0.31 mg ∙kg^−1^) (Fig. 2). The highest Co contents were found in the Kashubian and Masurian Lake Districts and the Eastern Sudetes (maximum 1.78 mg∙kg^−1^). The highest Cr contents were found in the Kashubian and Masurian Lake District (2.02 mg∙kg^−1^) and the Eastern Sudetes (maximum 9.73 mg∙kg^−1^). The highest Pb contents were found in the Silesian Lowland and the Central Sudetes (maximum 0.68 mg∙kg^−1^). The highest Ni contents were found in the Szczecin Coast, Wielkopolska Lake District, Niski Beskids and Żywiec Beskids (maximum 7.6 mg∙kg^−1^). The highest Zn contents were found in the Szczecin Coast, the Lubuskie and Wielkopolska Lake District, the Western Beskids and the Eastern Sudetes (maximum 47.35 mg∙kg^−1^).

The mean and maximum contents of most of the elements tested in pine needles from the peatlands in Poland were lower than those recorded at other sites in Europe, with only mean and maximum Cu, Ni and K being higher (Table 2). In the case of maximum Cu, this applied to two sites in the Lubuskie Lake District (10.14 mg∙kg^−1^) and the Silesian Lowlands (10.06 mg∙kg^−1^), while the Cu content was higher than the European mean at most peatlands (116) dispersed irregularly throughout Poland. The mean Ni at most peatlands (77) was higher than the European mean, while the maximum was higher at 64 of the sites studied (the highest in the Silesian Lowland: 8.19 mg∙kg^−1^). In the case of maximum K, this applied to one site in the Lubuskie Lake District (8313.70 mg∙kg^−1^), while the K content was higher than the European mean at almost half of the peatlands (48) irregularly distributed throughout the country.

**Table 2:** Content of selected chemical elements (mg∙kg^−1^) in one-year-old needles of Scots pine from peatlands in Poland compared to sites in Europe^a^ and norms for plants^b^.

Chemical element	Poland				Europe				Norms	
	Mean	Min	Max	sd	Mean	Min	Max	Sites	Normal level	Toxic level
Cd	0.19	0.03	0.98	0.15	0.57	0.03	1.53	10	0.01–5	5–30
Co	0.12	0.00	0.54	0.09	–	–	–	–	0.02–1	15–50
Cr	0.40	0.13	3.59	0.35	1.62	0.42	4.72	4	0.1–0.5	5–30
Cu	**5.13**	2.57	**10.14**	1.52	**4.23**	2.48	**9.82**	15	2–30	20–100
Pb	0.30	0.02	1.06	0.17	6.31	0.13	29.30	15	3–10	30–300
Ni	**2.04**	0.15	**8.19**	1.40	**0.91**	0.34	**1.41**	3	**0.1–5**	10–100
Zn	32.92	14.32	61.75	8.54	59.81	31.86	204.00	17	10–150	100–400
Al	89.11	14.36	191.16	36.33	237.33	157.00	363.00	6	–	–
Ca	1794.17	371.65	4677.27	686.62	3177.61	0.17	8000.00	14	–	–
Fe	35.99	20.47	118.72	13.11	131.63	31.00	618.00	13	–	–
K	**4919.48**	2331.44	**8313.70**	1168.88	**4909.17**	0.50	**8080.00**	13	–	–
Mg	780.43	456.04	1158.42	146.93	1115.48	790.00	1580.00	13	–	–
Mn	196.48	22.54	569.54	117.87	408.93	124.00	821.00	12	–	–
Na	21.14	6.94	58.66	7.28	117.96	26.12	461.00	10	–	–
P	1174.12	572.22	2234.38	303.77	1244.88	0.18	1780.00	14	–	–
C [%]	46.76	43.31	49.39	0.83	–	–	–	–	–	–
N [%]	1.30	0.73	2.00	0.24	–	–	–	–	–	–

^a^^11,15,17–25^, ^b^^3^.

Significant values are in bold.

The mean and maximum heavy metal content in pine needles from peatland did not exceed toxicity norms, with the exception of Ni at sites in the south part of Wielkopolska Lowland (5.46, 8.19 mg∙ kg^−1^), the Niski Beskids (6.81 mg∙kg^−1^), and the Lubuskie Lake District (6.20 mg∙kg ^−1^) was above the normal (optimal) level.

The highest concentrations of heavy metals such as Cd, Pb and Zn in pine needles were found in the Silesian Upland, with maximum values of 0.98, 1.06 and 61.75 mg∙kg^−1^, respectively (Fig. 3). The highest Co content was found in the western part of Pomeranian Lake District (0.54 mg∙kg^−1^), and Cr in the Silesian Highland (3.59 mg∙kg^−1^).

**Table 3:** Correlation coefficients (+ positive, − negative) between density index of selected geographical environment elements and chemical elements contained in one-year-old needles of Norway spruce and Scots pine. Significant (p < 0.05) relationships are marked in red.

Elements	Cd	Co	Cr	Cu	Pb	Ni	Zn	Al	Ca	Fe	K	Mg	Mn	Na	P	C	N
Water	−	+	−	−	−	−	−	−	−	+	+	+	−	+	+	+	+
Roads	+	−	+	−	**+ **	−	+	+	−	−	−	+	−	+	−	−	−
Railways	−	−	+	+	−	−	+	−	+	−	−	−	+	+	+	−	−
Power users	+	−	+	−	+	+	** +**	** +**	−	**+ **	+	**+**	−	−	−	−	**+ **
Population	+	−	+	−	**+**	−	** +**	+	+	**+**	+	**+**	−	+	−	+	** +**
Agriculture lands	−	−	+	+	−	+	−	+	+	−	−	**+**	−	−	+	−	+
Infrastructure	+	−	−	−	−	−	+	−	+	+	−	−	+	**+**	−	−	+
Human footprint index	+	−	+	−	+	−	+	+	+	+	+	+	+	+	+	−	+

The Mann–Whitney test showed that spruce and pine needles do not differ in the content of most of the elements studied, with the exception of Pb, Ni, Fe, C (p < 0.05). The Kruskal–Wallis test showed that spruce needles from Sapric Histosol have a significantly (p < 0.05) higher content of Cd and Mn than those from other types (Table 4). The Al content in the needles of this species was significantly (p < 0.05) higher on Hemic Histosol than on sapric, while the Ca content was higher on Sapric Histosol than on fibric. The contents of other elements in spruce needles did not differ significantly (p > 0.05) between Histosol types.

**Table 4:** Content (median ± quartile range) of selected chemical elements (mg∙kg^−1^) in one-year-old needles of Norway spruce from peatlands; a, b – homogeneous groups.

Histosol type			
Chemical element	Sapric	Hemic	Fibric
Cd	0.16 ± 0.12^a^	0.09 ± 0.08^b^	0.07 ± 0.08^b^
Co	0.13 ± 0.06^a^	0.14 ± 0.11^a^	0.1 ± 0.09^a^
Cr	0.46 ± 0.24^a^	0.47 ± 0.23^a^	0.5 ± 0.19^a^
Cu	4.25 ± 2.63^a^	4.05 ± 2.04^a^	4.18 ± 1.30^a^
Pb	0.23 ± 0.13^a^	0.26 ± 0.13^a^	0.26 ± 0.12^a^
Ni	1.63 ± 1.24^a^	1.55 ± 1.01^a^	1.43 ± 1.07^a^
Zn	25.39 ± 10.91^a^	24.28 ± 7.79^a^	24.47 ± 8.55^a^
Al	25.12 ± 17.16^a^	33.38 ± 21.55^b^	26.69 ± 21.71^ab^
Ca	2553.29 ± 956.09^a^	2363.34 ± 858.10^ab^	2168.08 ± 843.74^b^
Fe	33.40 ± 11.31^a^	33.41 ± 8.77^a^	32.41 ± 7.56^a^
K	5307.89 ± 2961.32^a^	5729.34 ± 2595.02^a^	5723.68 ± 2271.53^a^
Mg	659.84 ± 220.25^a^	665.7 ± 152.35^a^	708.07 ± 160.9^a^
Mn	128.3 ± 209.01^a^	265.84 ± 278.99^b^	242.03 ± 184.42^b^
Na	16.33 ± 7.58^a^	18.3 ± 12.66^a^	17.92 ± 8.82^a^
P	1449 ± 610.16^a^	1399.33 ± 658.28^a^	1391.31 ± 596.36^a^
C [%]	46.69 ± 0.65^a^	46.89 ± 0.86^a^	46.9 ± 1.20^a^
N [%]	1.19 ± 0.36^a^	1.17 ± 0.38^a^	1.1 ± 0.40^a^

Pine needles from Fibric Histosol had significantly (p < 0.05) higher Cr and K content than those from other Histosol types (Table 5). The Cu, P and N content in pine needles from Fibric Histosol was significantly (p < 0.05) higher than in Hemic Histosol, while the Ca content was significantly (p < 0.05) higher in Sapric Histosol than in Hemic Histosol. The content of other elements in pine needles did not differ significantly (p > 0.05) between Histosol types. Significant (p < 0.05), high (r > 0.5), positive correlation coefficients were found between Cu and Cr, K, P, N, and between Cr and Fe in spruce needles (Table 4). Cd and Ca contents significantly (p < 0.05) positively correlated with soil and water pH, while Mn content correlated negatively. Furthermore, Ni and Zn contents significantly (p < 0.05) positively correlated with water pH, while C content correlated negatively.

**Table 5:** Content (median ± quartile range) of selected chemical elements (mg∙kg^−1^) in one-year-old needles of Scots pine from peatlands; a, b – homogeneous groups.

Histosol type	Sapric	Hemic	Fibric
Chemical element			
Cd	0.17 ± 0.15^a^	0.13 ± 0.09^a^	0.17 ± 0.1^a^
Co	0.11 ± 0.09^a^	0.08 ± 0.07^a^	0.11 ± 0.09^a^
Cr	0.32 ± 0.07^a^	0.31 ± 0.12^a^	0.40 ± 0.18^b^
Cu	4.64 ± 0.26^ab^	4.50 ± 1.85^a^	5.39 ± 1.81^b^
Pb	0.28 ± 0.2^a^	0.26 ± 0.15^a^	0.26 ± 0.26^a^
Ni	1.87 ± 1.78^a^	1.73 ± 1.74^a^	1.62 ± 1.71^a^
Zn	31.11 ± 11.1^a^	30.27 ± 9.85^a^	33.33 ± 10.07^a^
Al	73.52 ± 60.32^a^	88.84 ± 51.61^a^	94.49 ± 40.54^a^
Ca	2231.63 ± 551.58^a^	1604.72 ± 849.8^b^	1659.99 ± 719.86^ab^
Fe	32.29 ± 6.72^a^	31.08 ± 8.42^a^	35.26 ± 9.56^a^
K	4230.64 ± 950.6^a^	4487.5 ± 1511.53^a^	5498.55 ± 1164.75^b^
Mg	716.34 ± 128.87^a^	788.65 ± 184.27^a^	756.05 ± 152.32^a^
Mn	158 ± 149.61^a^	157.37 ± 132.58^a^	199.46 ± 170.39^a^
Na	20.56 ± 8.26^a^	19.43 ± 7.67^a^	20.61 ± 8.1^a^
P	1241.91 ± 366.26^ab^	1092.24 ± 371.11^a^	1232.77 ± 316.69^b^
C [%]	46.48 ± 1.21^a^	46.88 ± 0.78^a^	46.71 ± 0.92^a^
N [%]	1.22 ± 0.22^ab^	1.15 ± 0.34^a^	1.44 ± 0.24^b^

**Fig. 4:**
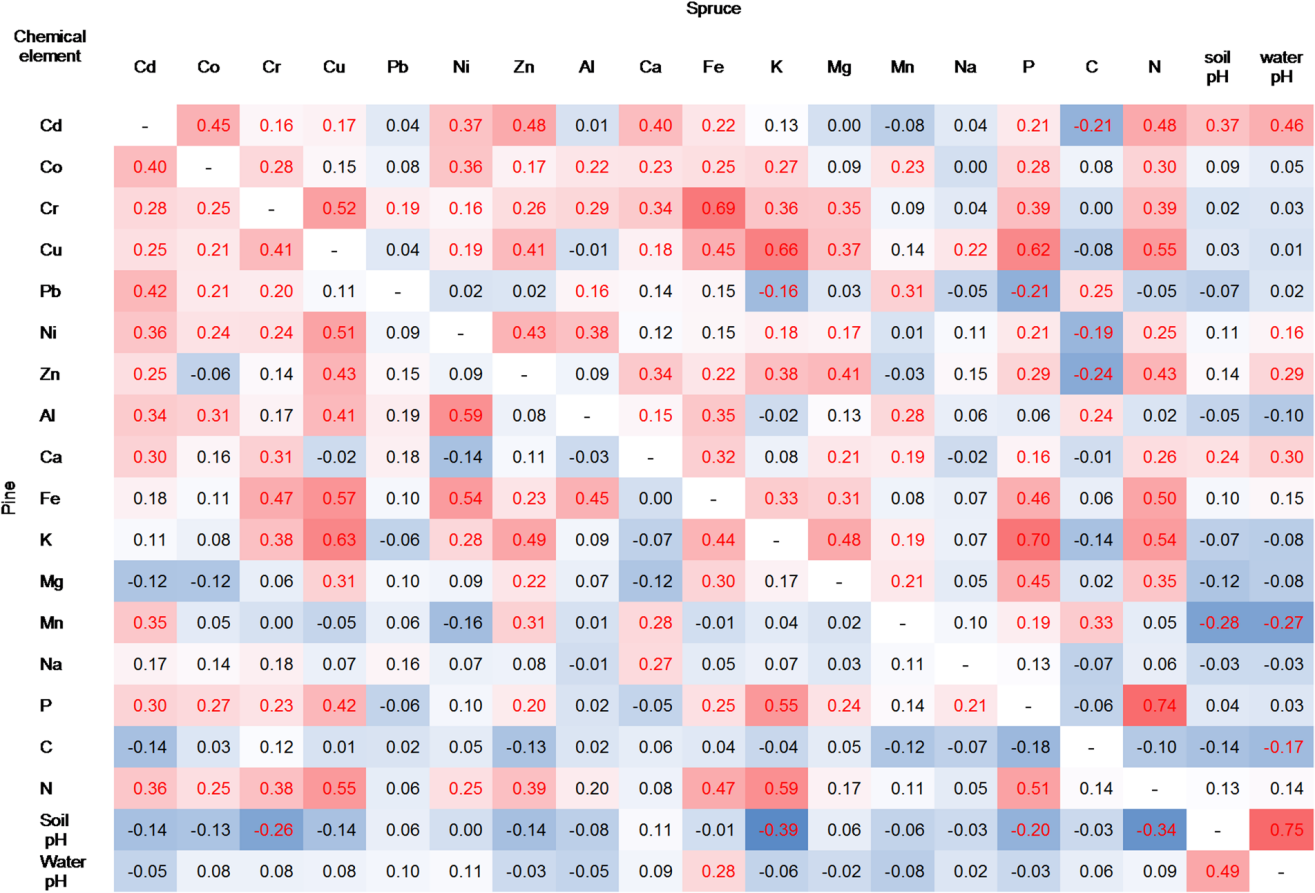
Correlation coefficients between selected chemical elements contained in one-year-old needles of Norway spruce (upper half) and Scots pine (lower half) and soil and water pH. Significant (p < 0.05) values are marked in red.

Significant (p < 0.05), high (r > 0.5), positive correlation coefficients were found between Cu and Cr, K, P, N, and between Cr and Fe in spruce needles (Fig. 4). Cd and Ca contents significantly (p < 0.05) positively correlated with soil and water pH, while Mn content correlated negatively. Furthermore, Ni and Zn contents significantly (p < 0.05) positively correlated with water pH, while C content correlated negatively.

Significant (p < 0.05), high (r > 0.5), positive correlation coefficients were found between Cu and Ni, Fe, N, between Ni and Al, Fe, between K and P, N, and between P and N in pine needles (Fig. 4). The contents of Cr, K, P, N correlated significantly (p < 0.05) negatively with soil pH, while Fe content correlated positively with water pH.

**Fig. 5:**
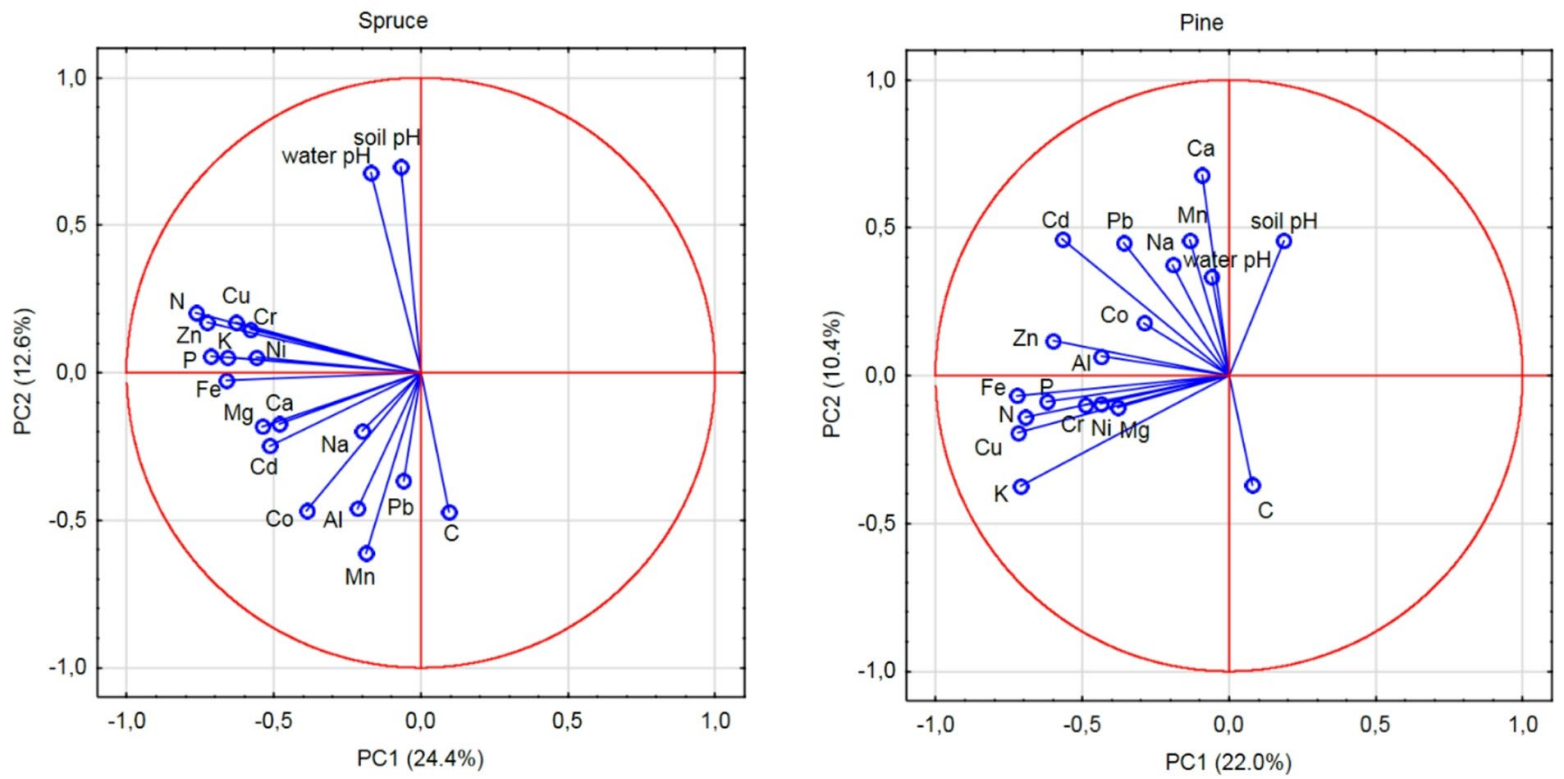
Projection of chemical elements contained in one-year-old needles of Norway spruce and Scots pine, and soil and water pH (variables) relative to the first (PC1) and second principal component (PC2).

PCA analysis of the chemical element content in spruce needles divided them into two groups in relation to PC2 (Fig. 5). The first group included Cu, Cr, K, P, N, Ni, Zn, and the second group included Cd, Co, Pb, Al, Ca, Fe, Na, Mg, Mn. In the first group, Cu, Cr, Zn, N and also Ni, P, K correlated most strongly positively with each other, while in the second group, Cd, Ca, Mg correlated most strongly positively with each other.

PCA analysis of the element content in pine needles identified two groups in relation to PC2 (Fig. 5). The first group included Cd, Co, Zn, Pb, Al, Ca, Na, Mn, and the second group included Cr, Cu, Ni, Fe, K, N, Mg, P. In the first group, Zn and Al correlated most strongly positively with each other, while in the second group, Cr, Ni, N, Cu, Mg correlated most strongly positively with each other. The C content in the needles of both spruce and pine correlated negatively with soil and water pH.

## Discussion

Combustion processes in industry, as well as in the municipal and residential sectors, are by far the dominant source of heavy metal emissions. Emitters in these sectors are predominantly located in the more densely populated southern part of Poland. The volume of heavy metal deposition in the soil from local emitters should be supplemented by their cross-border flow with prevailing winds from the western and southern sectors^8^. During long-distance transport of heavy metals in the air, their content decreases with distance from the emitters^9^, which was well reflected in the results of studies on heavy metal concentrations in the soils of forest reserves in the Silesian Province conducted by Staszewski et al.^10^. However, the results of our research indicate a large and undirected spatial variability of heavy metals and biogenic elements in the needles of Norway spruce and Scots pine in peatlands in Poland. Their content was not clearly related to southern regions with high: industrialisation, urbanization, population density and altitude above sea level. This does not correspond with the results of Staszewski et al.^14^, who found that the content of heavy metals accumulated on the surface of needles was generally higher in parks in the southern part of the country, and the highest heavy metal load indices were recorded in mountain national parks located in south-western Poland.

The analysis of the content of most elements in spruce and pine needles from peatlands in Poland indicates lower mean and maximum values compared to literature data from other regions of Europe. These results may indicate a relatively low level of atmospheric pollution deposition on the assimilation apparatus in the studied ecosystems, which was also noted in the soil of the studied peatlands^32,33^. Due to their sorption properties and isolation from intensive human activity, peatlands are often characterised by a lower human impact^3^.

Exceptional results were obtained for copper in the peatlands of north-western (spruce) and western (pine) Poland, where its concentration in needles exceeded the values recorded in other European countries. An increase in heavy metal emissions in Poland was recorded only for copper in relation to 1990 and 2005. Copper emissions mainly originate from tribological processes (friction processes) in road and rail transport, and their increase is mainly related to the increased number of road vehicles and their transport operations^8,35^. This fact may explain the high Cu content in peatlands in the vicinity of national roads and railway tracks. Increased Cu levels may also be associated with local emission sources, the use of fertilisers and plant protection products^4^. Copper concentrations in spruce needles above the European average (i.e. > 5 mg·kg^−1^) are also found in about two-thirds of Slovakia. The highest Cu values were found in the Spiš region^12^. Normal (optimal) levels for tree foliage, according European norm^11^, have been exceeded for Cu in the Western Carpathians, Eastern Carpathians and Southern Carpathians^12^. At the parallel, it should be noted that copper, as an element essential for the proper functioning of plants, does not always lead to negative physiological effects at higher concentrations, although it may limit the uptake of other micronutrients. Copper is essential for plants for proper metabolism at both the cellular and whole-organism levels as a component of many enzymes, proteins and chlorophyll^3^.

In the context of environmental risk assessment, it is important to note that the heavy metal content in needles did not exceed general toxicity standards, with the exception of Cr in one peatland with spruce in the Żywiecki Beskids (south-east slope of Babia Góra). Nickel was present at levels above the optimal range for conifers, which may indicate potential metal stress and metabolic disorders, including photosynthesis and water management^36^. Although these values do not indicate a direct threat to the studied peatlands, they may signal local emission sources or specific habitat conditions. Arndt et al.^37^ observed very increased levels of cadmium in four national parks: Magurski, Babiogórski, Ojcowski and Poleski, which corresponds to our results only in the first two regions mentioned. Cadmium plays no vital role in plants. It is a typical passive pollutant^3,38^. Its excess manifests itself in root shortening, dark green colouration and leaf wilting^3^. Above and below a certain limit, the concentration of essential nutrients causes deterioration in the health of forest trees and disrupts the circulation of elements in the ecosystem^12^. In our work, this may apply to increased potassium in pine needles in almost half of the studied peatlands irregularly distributed throughout the country.

We did not find many significant differences (p < 0.05) in the element content in spruce and pine needles growing at Fibric, Hemic and Sapric Histosol, nor did we find a significant (p < 0.05), high (r > 0.5) correlation between the elements in needles and the pH of water and soil. Only high cadmium and manganese content in spruce needles was found in Sapric Histosol, and high chromium and potassium content in pine needles was found in Fibric Histosol. Our results do not correspond with the findings that heavy metal bioaccumulation is related to pH, moisture content and the degree of organic matter decomposition in the soil^3,33,39–41^. Thus, they confirm that heavy metals mainly enter needles passively through stomata in the form of gas, rainwater solution, or dust accumulation on their surface^9,10^, while their active uptake from soil solutions through roots appears to be marginal. The concentration of elements in tree needles also depends on their age, e.g. nickel and lead are more abundant in older needles, while copper and cadmium are more abundant in younger needles^42^.

Additional heavy metal delivery through air pollution causes synergistic and antagonistic reactions. The mutual correlation between N, P, K, Ca, Mg in several dozen plant species was first described by Markert^43^. Potassium and nitrogen are important in protein biosynthesis, while calcium and magnesium are common enzyme activators in physiological metabolic processes^12^. Our PCA analysis of elements in spruce needles showed that K and Ni correlated most strongly with P, while N correlated most strongly with Cu, Cr, Zn, Ca, and Mg correlated most strongly with Cd. In the case of pine, the relationships between the elements most important for physiological processes and heavy metals were not clear. Staszewski et al.^14^ proposed cadmium as a marker reflecting the impact of industrial emissions on forests, as it correlates significantly with all heavy metals. Our results, both from the correlation analysis and PCA of element concentrations in needles, indicate that chromium appears to be such a marker for spruce-covered peatlands, while Cd could be a marker for pine-covered peatlands.

## Conclusions

The results of our research indicate that the spatial variation in heavy metal content in the needles of Norway spruce and Scots pine in peatlands in Poland is not clearly related to the human footprint index. This suggests a probable influence of episodic local sources of pollution and transboundary transport of air pollutants on their bioaccumulation. They support the view that even relatively remote or less industrialized areas are not isolated from human impacts, as pollutants can be transported over long distances across national boundaries. Consequently, effective environmental monitoring and mitigation strategies require not only local or national approaches but also international cooperation and integrated policies addressing transboundary air pollution.

Increased copper concentrations in spruce and pine needles in Polish peatlands, especially in the north-western and western regions, indicate an important impact of emissions from road and rail transport. However, despite exceeding the optimal values for some elements, the total heavy metal levels do not pose a toxicological risk, but may indicate local biogeochemical disturbances and metallic stress in plants. These findings highlight the growing role of diffuse and transport-related emission sources in shaping the chemical status of ecosystems, even in areas not directly adjacent to major industrial centers. They also reflect a global trend in which reductions in point-source industrial pollution are increasingly accompanied by the relative importance of mobile emission sources, such as traffic, in contributing to trace metal deposition.

The content of elements in spruce and pine needles in peatlands in Poland does not significantly depend on the type of Histosol or the pH of water and soil, which indicates that heavy metals accumulate mainly passively from the atmosphere, and active uptake by roots plays a marginal role, although the concentrations of some elements also depend on the age of the needles, as reported in other studies. This underlines the necessity of considering atmospheric deposition as a major component in global environmental assessments and reinforces the importance of coordinated international monitoring of air quality.

Significant correlations between chromium and cadmium heavy metals and certain heavy metals and biogenic elements in tree needles indicate that the degree of environmental pollution affects plant metabolic relationships, and mobile elements such as chromium in spruce and cadmium in pine can serve as markers of industrial emissions’ impact on peatland ecosystems. These heavy metals may try using in large-scale environmental assessments for coordinated international monitoring of ecosystem. However, when interpreting the results, one should take into account the natural variability in the chemical composition of needles, the seasonality of sampling, the specificity of peatlands as habitats with different hydro-chemical conditions and no studied transboundary transport of air pollutants.

## Data Availability

Data will be made available on request.
